# Operational Challenges in the Use of Structured Secondary Data for Health Research

**DOI:** 10.3389/fpubh.2021.642163

**Published:** 2021-06-15

**Authors:** Kelsy N. Areco, Tulio Konstantyner, Paulo Bandiera-Paiva, Rita C. X. Balda, Daniela T. Costa-Nobre, Adriana Sanudo, Carlos Roberto V. Kiffer, Mandira D. Kawakami, Milton H. Miyoshi, Ana Sílvia Scavacini Marinonio, Rosa M. V. Freitas, Liliam C. C. Morais, Monica L. P. Teixeira, Bernadette Waldvogel, Maria Fernanda B. Almeida, Ruth Guinsburg

**Affiliations:** ^1^Escola Paulista de Medicina, Universidade Federal de São Paulo, São Paulo, Brazil; ^2^Fundação Sistema Estadual de Análise de Dados, São Paulo, Brazil

**Keywords:** public health, datasets as topic, population studies in public health, Death Certificates, Birth Certificates, secondary health data

## Abstract

**Background:** In Brazil, secondary data for epidemiology are largely available. However, they are insufficiently prepared for use in research, even when it comes to structured data since they were often designed for other purposes. To date, few publications focus on the process of preparing secondary data. The present findings can help in orienting future research projects that are based on secondary data.

**Objective:** Describe the steps in the process of ensuring the adequacy of a secondary data set for a specific use and to identify the challenges of this process.

**Methods:** The present study is qualitative and reports methodological issues about secondary data use. The study material was comprised of 6,059,454 live births and 73,735 infant death records from 2004 to 2013 of children whose mothers resided in the State of São Paulo - Brazil. The challenges and description of the procedures to ensure data adequacy were undertaken in 6 steps: (1) problem understanding, (2) resource planning, (3) data understanding, (4) data preparation, (5) data validation and (6) data distribution. For each step, procedures, and challenges encountered, and the actions to cope with them and partial results were described. To identify the most labor-intensive tasks in this process, the steps were assessed by adding the number of procedures, challenges, and coping actions. The highest values were assumed to indicate the most critical steps.

**Results:** In total, 22 procedures and 23 actions were needed to deal with the 27 challenges encountered along the process of ensuring the adequacy of the study material for the intended use. The final product was an organized database for a historical cohort study suitable for the intended use. Data understanding and data preparation were identified as the most critical steps, accounting for about 70% of the challenges observed for data using.

**Conclusion:** Significant challenges were encountered in the process of ensuring the adequacy of secondary health data for research use, mainly in the data understanding and data preparation steps. The use of the described steps to approach structured secondary data and the knowledge of the potential challenges along the process may contribute to planning health research.

## Introduction

Secondary health data supports information production to develop and evaluate preventive and therapeutic strategies, services, programs, and health policies. It is quite advantageous to be able to use these data for research purposes since they have been already collected (1–3).

In Brazil, social and health data, collected continuously or periodically, are, in general, structured (variables with previously established meaning and coding), consolidated, anonymized, and with unrestricted public access (4–11). Along with the data, the distribution agencies also make the materials available for their understanding, such as operational manuals, dictionaries of variables, and models of collection instruments, as well as tools for their visualization in the form of graphs, tables, or maps (7–11).

In the United States, for example, the Center for Disease Control and Prevention (CDC) internet site makes a lot of structured secondary health data publicly available (12, 13), and also provides restricted access data for research (14), and tools to query the data (13). Data from other countries can also be found at http://ghdx.healthdata.org/ (15).

Some of the Brazilian agencies that provide open data are the Interagency Health Information Network (RIPSA) (6), the Information Technology Department of the Public Health Care System (DATASUS) (7), the Brazilian Institute of Geography and Statistics (IBGE) (8), the São Paulo State Data Analysis System Foundation (SEADE) (9) and the Brazilian Open Data Portal (10, 11).

Considering the whole country, the main source of secondary health data is DATASUS (7). Such available health data are collected through DATASUS (7)'s Information Systems and stored in administrative databases. The use of secondary health data such as those from DATASUS (7) databases has become increasingly frequent as can be seen, searching the PubMed (MEDLINE) database under the query “datasus (Title/Abstract).”

Among the DATASUS's Information System, the Mortality Information System (SIM) and the Live Birth Information System (SINASC), stand out for their importance in the generation of vital statistics and social indicators, living conditions, and child health (7, 16–19). These 2 databases contain information on all live births and all deaths informed in the whole country, independently if an individual is a user of the Brazilian public health care or not.

Live births and death data are collected in the Live Birth Certificates (LBC) and Death Certificates (DC), respectively. The paper forms, LBC and DC, are completed in three copies: (a) feeds the national database SIM and SINASC; (b) is retained in the Civil Registry office in which the birth or death event is registered and (c) is retained in the service informing the event (9).

In the State of São Paulo (SP), SEADE Foundation is the institution responsible for the collection, organization, analysis, and dissemination of these records (death and live births) under which the vital statistics of SP are produced from data present in LBC and DC (9). The Civil Registry offices of 645 municipalities in SP send the completed LBC and DC forms to SEADE monthly. After being entered into SEADE's system, infant death data (infant under 1 year of age) is linked to birth data (9). The linked file consists of deaths of infants born in a given year including their birth variables. While anonymized births and death records are publicly accessible on an internet site, linked files are not.

A similarly linked dataset of live births and infant death who died in the United States, Puerto Rico, The Virgin Islands, and Guam are available as downloadable data files on the internet site https://www.cdc.gov/nchs/data_access/vitalstatsonline.htm. The linked birth and infant death dataset is also available in birth cohort data format, with the complete description of these data (20).

In scientific research, the concomitant use of these data makes it possible to calculate the risk estimates of infant death and its age components, to analyse risk factors or determinants of specific outcomes, to estimate cause-specific mortality rates, and to analyze time series and spatial and ecological studies (2, 16, 21–23).

Despite this favorable and stimulating scenario, secondary data is not always ready for use. In these situations, there are difficulties to be considered, such as limiting the data to certain geographic areas or periods, when there are changes in the way of collecting the variables, lack of standardization in the data format, discontinuity in the collection of some data over time or variation in coverage (24). Besides, it may be necessary to select them according to conditions related to the inclusion or exclusion criteria, which is done by transforming data from the original database to obtain the study population. Anyway, there are many situations, conditions, or factors that can impact the usability of the data for purposes other than those for which it was collected. To answer the research questions, the data must be organized in a way that their handling is quick and easy. However, depending on the resources available, these operations may not be easily implemented.

The knowledge of the steps and obstacles that can arise using secondary data for specific purposes in research potentially allows to identify critical points, and, consequently, to plan actions and direct resources for the effective execution of research projects. Thus, this study aimed to describe the steps necessary to ensure an adequate set of structured secondary health data for use in quantitative research and to identify the challenges of this process. The data will be considered adequate for use if they fit the purpose of the research; and are ready to be used in the planned analyses, and there are no ethical constraints in using them.

## Materials and Methods

The present study is a qualitative study that reports methodological issues related to the use of a secondary health database.

The study material consisted of all records of live births between 2004 and 2013, and infant deaths (0–365 days) of children born from mothers residing in one of the 645 municipalities in the State of São Paulo, totaling 6,059,454 births and 73,735 deaths. These records were originated in the Civil Registry Offices and made available in digital format by SEADE for the execution of a project on neonatal mortality. These secondary data were called “input data” (25–27). These data were anonymized, following Brazil's General Data Protection Law, which has respect for privacy as its basic principle (28).

This study is part of a project on neonatal mortality carried out at the Federal University of São Paulo and was approved by the Research Ethics Committee of the institution under opinion 2.580.929 of 08/04/2018. The referred project is entitled “Secular trend, spatial evolution and maternal and neonatal conditions associated with early and late neonatal mortality due to respiratory disorders, infections, congenital anomalies and perinatal asphyxia in the state of São Paulo between 2002-2015.”

Data should be prepared for a cohort study in which live births would be classified into two groups: those who died between 0 and 27 days and those who were alive until the 27th day of life. For deaths, data come from SEADE Foundation's database of “Death linked to birth.” The linked file consists of death records of infants born in a given year including their birth variables. All planned analyses would be made according to the cause of death and age of death (1st hour, 1st 24 h, 0–6 days, and 7–27 days after birth).

In this study, the adequacy of secondary data for use in research refers to the potential of the data set to meet planned analysis needs, that is, the dataset is ready for starting data analysis. In this context, the approach of the present study was to divide the data adequacy process into steps, following their function.

**Figure 1:**
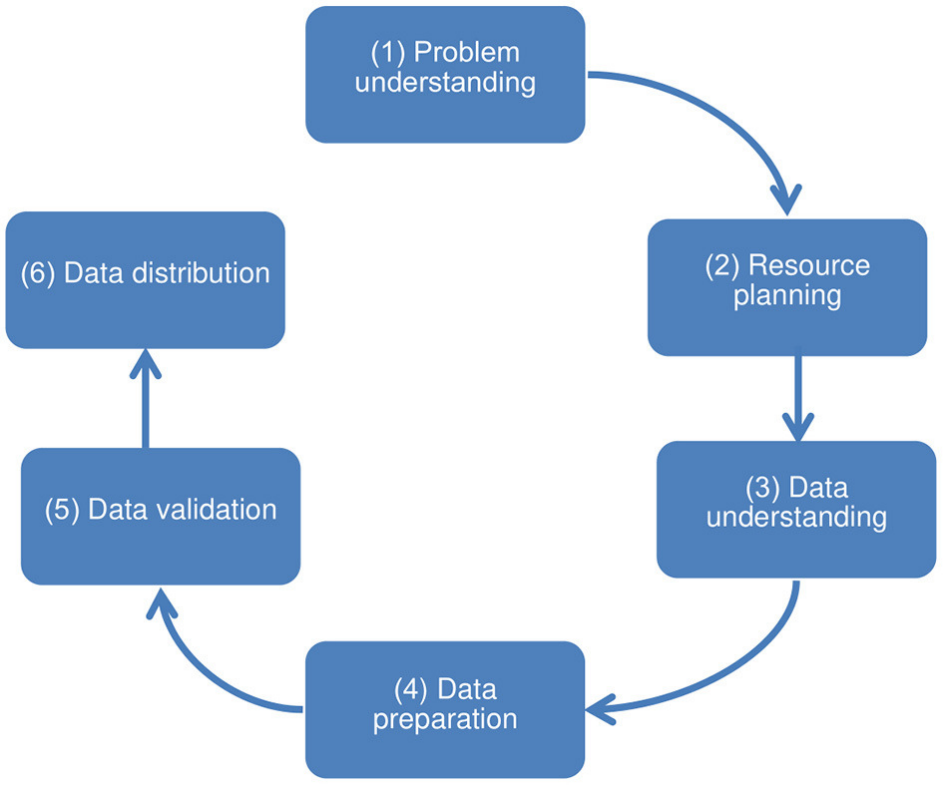
Steps toward ensuring the adequacy of structured secondary health data for use in quantitative research.

Initially, the data adequacy process was based on the first three steps of the CRISP-DM (Cross Industry Standard Process for Data Mining) data science technique (29, 30), which precede the data analysis: problem understanding, data understanding, and data preparation. Also, it was necessary to include three other steps to organize other procedures and operational challenges that went beyond this scope. Thereby, the challenges and description of the procedures to ensure data adequacy were undertaken in 6 steps: (step 1) problem understanding, aimed at understanding the use of data and surveying its characteristics; (step 2) resource planning, aimed at human resources, hardware, and software for subsequent steps; (step 3) data understanding, aimed at collecting and understanding the meaning and organization of the input data; (step 4) data preparation, intended for handling input data and making the output data set; (step 5) data validation, intended for data homologation; and (step 6) data distribution, for the delivery of approved output data, prepared for the specific use and ready for handling. The sequence of steps is presented as a non-cyclical path since moving back to previous steps is not expected (Figure 1).

For the execution of all steps, periodic meetings were planned with the participation of 12 researchers, users of the final data, who formed a working group composed of professionals in the areas of healthcare (doctors and physiotherapists) and Formal Sciences (computing and statistics).

The main research questions are sufficiently defined above. In summary, the premises of this study are: secondary data are not ready for the intended use; there is evidence of the relevance of these data for quantitative research purposes; and the adequacy of the data cannot be determined effectively with the use of simple tools which are commonly used for data preparation.

Based on these premises and the established steps, procedures were planned to ensure the adequacy of the study material for the intended use, and as defined by the working group.

In the execution of the procedures, the operational challenges encountered and the actions to face them were observed and recorded. The existence of any barrier to the use of data was considered an “operational challenge.”

For each step, procedures, challenges encountered, actions to cope with them, and partial results were described. To identify the most labor-intensive tasks in this process, the steps were assessed by adding the number of procedures, challenges, and coping actions. The highest values were assumed to indicate the most critical steps.

## Results

Table 1 presents the 22 procedures distributed in the 6 steps of the adequacy process of structured secondary health data for specific uses in quantitative research. The steps of problem understanding, data understanding, and data preparation stood out concerning the quantity with 5, 7, and 6 procedures, respectively.

**Table 1:** Procedures at each step for ensuring the adequacy of structured secondary health data for specific use in quantitative research.

**Step**	**Procedures**
1. Problem understanding	1. Assessment of the characteristics of secondary input data
	• Content: what the data represents in the real world, source of data, the context in which it was collected
	• Estimated volume: number of records and size of expected files
	• Expected data file format
	2. Assessment of the characteristics of the research
	• Population and period under study
	∙Inclusion and exclusion criteria for selection
	• Study design and analysis unit
	• Variables involved in the main research questions, objectives, and hypotheses
	3. Assessment of the characteristics of the output data
	• Estimated output data volume: number of records or file size
	• The desired format for delivery of output data
	4. Checking the availability of input data and variable dictionaries
	5. Evaluation of the ethical aspects and technical feasibility of data adequacy for the research
2. Resource planning	6. Sizing up human resources
	7. Sizing up computational resources (hardware and software platform)
	• Volume and format of input data
	• Support for the operations required to adjust the input data
	• Estimated volume and format of output data
	• Performance and data volume limits for eligible computing resources
3. Data understanding	8. Obtaining secondary data files and variable dictionaries
	9. Understanding the variable dictionaries related to the input data and creating the research variables dictionary for each type of file
	10. Inventory of data files: name and extension, size in bytes, and number of records
	11. Assessment of the existence of a unique record identifier (primary key) in each data file
	12. Inventory of the variables contained in the data files: name, type, and size
	13. Exploratory data analysis for completeness
	14. Elaboration of the data extraction plan for the research
4. Data preparation	15. Execution of the data extraction plan
	16. Exploratory data analysis to detect invalid content and assess the homogeneity in a data filling
	17. Data cleaning and transformation to generate research variables
	18. Updating the search variable dictionary
5. Data validation	19. Exploratory analysis of the transformed data for comparison with the original data
6. Data distribution	20. Exporting the database to the specified format (s)
	21. Reduction of the database to contain only the research variables
	22. Distribution of the database and dictionary of research variables

Table 2 presents the operational challenges encountered and the actions to face them. A total of 27 operational challenges were identified, of which 66.7% (18 from 27) were from the steps of data understanding and data preparation (steps 3 and 4), corresponding to operational challenges 6–23. In these steps were also concentrated most actions to face these challenges (15 from 23), coping actions 5–19.

**Table 2:** Operational challenges identified in the steps for ensuring the adequacy of structured secondary health data for specific use in quantitative research.

**Step**	**Operational challenges**	**Coping actions**
1. Problem understanding	1. Unavailability of the complete set of data files for immediate access 2. Lack of definition on how to access the variable dictionary 3. Interdisciplinary communication in the team 4. Establishment of consensus in the decisions and definitions	1. Meetings with the institution providing the data 2. Recording of decisions and definitions 3. Obtaining a sample of the data to assess the technical feasibility of data adequacy for the specific use
2. Resource planning	5. Need to optimize cost and preparation time for a large volume of data (~10 Gigabytes) in more than one format	4. Prioritizing the use of available human and computational resources and planning the acquisition of complementary computational resources to minimize the training time for human resources
3. Data understanding	6. Need to improve understanding of variables 7. Variables that have changed their format over time 8. Multiple files with different structures 9. No unique identifiers of records 10.File structure differ from data dictionary description 11. Variables filled with codes from other information systems	5. Consultation with other sources of information and exchange of information in periodic meetings 6. Storage of data in database tables, using text fields 7. Unique identifier insertion of records to make them logically accessible 8. Log and reuse of commands (queries) in SQL language when possible 9. Making variable dictionaries with standardized names 10. Elaboration of the data extraction and combination plan: reduction of the number of tables; standardization of data structures; adding the source in the primary key of the tables; and identification of variables to filter the records of interest
4. Data preparation	12. Multiple tables 13. Multiple values to denote Null content 14. Different filling formats in date variables 15. Invalid values 16. Different filling formats in numeric variables 17. Variables with mixed content 18. Variables with multiple contents 19. Duplicates in variables with multiple contents 20. No rules for cross consistency of related variables 21. Variable filled with code dependent on an external database 22. No direct reference to the external databases used 23. No single variable for data file integration	11. Reducing the original data (multiple tables) to two tables (table union) 12. Extraction of records of interest after combining data 13. Standardization of null content and recount of nulls 14. Elaboration and execution of the cross-consistency rules of the variables 15. Standardization of variable formats 16. Search for official databases to decode variables dependent on external codes 17. Incorporation of the description of external codes in the research database 18. Separation of variables with multiple contents into new variables for decomposition into single content 19. Data integration using a set of variables common to the tables
5. Data validation	24. No single report with the same scope in the original data source for comparison with prepared data	20. Validation of the transformed data based on the expected data volume and the frequency distribution of each variable according to a time dimension
6. Data distribution	25. Big data volume (approximately 10 Gigabytes) 26. Need to deliver output data in more than one format 27. Need for storage and backup of work files	21. Use of a statistical package to incorporate the dictionary of variables into the data 22. Use of converter software to export data and variable dictionary 23. Creation of private cloud for data distribution and users with different access levels

Table 3 shows the results achieved after the execution of the procedures and actions to face the operational challenges corresponding to each step. In one case due to the need to create a new variable not previously defined, it was necessary to return to the data preparation step.

**Table 3:** Results achieved at each step for ensuring the adequacy of structured secondary health data for specific use in quantitative research.

**Step**	**Results achieved**
1. Problem understanding	*Definitions and Information acquired*
	• Input data: annual records of infant deaths (~8 thousand) and live births (~600 thousand) in Microsoft® Excel spreadsheet format (*.xlsx*) (41); deaths deterministically linked to births (25, 26), with coded diagnoses (42); and variable dictionaries available (43)
	• Output data: organized for the cohort study with neonatal death as the main outcome (~70% of infant deaths); suitable for the selection of specific causes of death; with group identification and classification of cases of congenital anomaly in death or live birth
	• Distribution file format: flat-file (.*csv*) and variable dictionaries in Microsoft® Word 2010 (41) format (*.docx*); data and variable dictionary embedded in data file format of the statistical packages SPSS v24® (44) *(.sav)* and Stata v15® (45) (*.dta*)
2. Resource planning	*For steps 3, 4 and 5*
	• Hardware: portable computer with 16 gigabytes of RAM (random access memory) and 1 terabyte hard disk
	• Operating System: Microsoft® Windows 10® (46)
	• Software: Microsoft® Office Professional 2010® (41), Microsoft® SQL Server 2012 Express® (34), SPSS v24® (44) and Stat Transfer v14® (47)
	• Human Resources *(Peopleware)*: training in SQL language
	*For step 6*
	• Platforms: Cloud storage system (OwnCloud version 10.5.0), free and open source running on a virtual machine based on the Linux operating system (Fedora Server 31, kernel 5.7.15), maintained as a Virtual Machine in the Research Datacenter of the Federal University of São Paulo (https://www.dis.epm.br/#/parque_maquinas)
	• Peopleware: Network and infrastructure analyst
3. Data understanding	*Stored, understood and identified data*
	• Anonymized annual data has been imported into 20 tables
	• Infant death files contained variables present in the Death and Live Birth Certificates
	• The live birth files contained variables from Live Birth Certificates
	• The causes of death were reported in 6 variables (basic cause, line A, line B, line C, line D, line II) and the lines could contain one or more International Classification of Diseases codes, 10th Revision (42)
	• Information on anomalies was present in the records of Death and Live Birth Certificates
	• Sequential identification has been added as a primary key in the tables
	*Data files extraction and merging plan*
	• Data Merging (Combination of input data) to reduce tables and include data source identification for the primary key composition
	• Data Extraction based on two variables: age and municipality of residence
4. Data preparation	*Combined and extracted data*
	• Data reduced to 2 tables with defined primary key and unified variables formatted as text type
	• 50,842 neonatal deaths were extracted, without changing the number of live birth records
	*Transformed and integrated data*
	• Standardization and cleaning of data, observing the presence of invalid values; invalid formats of data type variables; variables filled in as number and text; possibility to correct the format; errors revealed by crossing related variables
	• Integration of the two tables for the cohort study using the common variables: 50,247 neonatal death records were identified among the Live Birth Certificates
	• The causes of death were stored in 27 new variables and the duplicates were eliminated
	• Cases of congenital anomaly have been identified; diagnoses of anomaly in live births were organized into 10 new variables; deaths with anomaly were classified into 11 groups (present or absent)
	• The descriptions of the external codes were incorporated into the data: diagnoses (42) and the municipalities (IBGE) (48)
5. Data validation	*Validated data and the completed dictionary of variables*
	• Counting the total number of neonatal deaths and exploring the main variables per year
	• The need to create a new variable was identified, going back to the previous step
	• Database was approved and the dictionary of variables was finalized
6. Data distribution	*Data arranged in the specified and distributed formats*
	• Data exported from MS® SQLServer® (34) in .csv format and imported in the SPSS® (44) statistical package (.sav)
	• The description of the variables and their values has been incorporated into the data file (.sav)
	• .sav file has been converted to the Stata® statistical package data file format (.dta) (45, 47)
	• Cloud storage (https://doc.bioinfo.unifesp.br/cloud) for sharing or distribution

Table 4 summarizes the number of procedures (detailed in Table 1), operational challenges (detailed in Table 2), coping actions (detailed in Table 2), and the criticality ranking for each step. Starting with the most critical step, the resulting ranking was: (1st) data preparation; (2nd) data understanding; (3rd) problem understanding; (4th) data distribution; (5th) resource planning; and (6th) data validation.

**Table 4:** Number of procedures^*^, operational challenges^**^, coping actions^**^ and criticality ranking of the steps for ensuring an adequate set of structured secondary data in the health field for specific use in quantitative research.

**Step**	**Procedures**	**Operational challenges**	**Coping actions**	**Critical order**
1. Problem understanding	5	4	3	3
2. Resource planning	2	1	1	5
3. Data understanding	7	6	6	2
4. Data preparation	4	12	9	1
5. Data validation	1	1	1	6
6. Data distribution	3	3	3	4
Total	22	27	23	–

* *The procedures are listed in Table 1*,

** *operational challenges and coping actions in Table 2*.

The size of files and number of records processed are presented in Supplementary Material 1. The data description of the final dataset is presented in Supplementary Material 2.

In the research project for which the data were prepared, the objectives, study design, and the intended use of the data were clearly defined, but they had not been sufficiently detailed for the elaboration of the research database, a task that was done only after receiving the data or a sample of it, in step 1 (Tables 1–3). The tasks in step 1 help to refine the data usage needs that were often not sufficiently clear in the research project or that have changed. For example, in the present study, initially, to classify a death with congenital anomaly, it was expected to involve only checking if one of the causes of death was in the code range (Congenital anomalies: Q0–Q99). Reviewing the needs of the project, it was realized that it was also necessary to classify individuals into groups of anomalies, being that the same individual could present anomalies of one or more groups (Table 3, Supplementary Material 2).

After performing the procedures in step 1- Table 1, it was possible to observe that the data were not prepared in an adequate way for the intended use. For example, the input data were distributed in several files with different structures. Therefore, in order to represent the cohort design study in a flat-file, the birth data should be combined with the “death linked to birth” data. It was also necessary to classify all live births in two groups, according to neonatal death outcome, as declared on the referred research project.

The procedures showed in Table 1 and the coping actions of the challenges listed in Table 2 were necessary tasks to prepare these input data for the intended use.

The execution of the procedures and actions addressing the challenges in each step resulted in a database which was ensured for adequacy in its intended use, according to the defined requirements in step 1. According to the definitions regarding the contents and file formats of the output data, the final product of the described process was a simple file, distributed in 3 formats, containing the consolidated data and integrated to represent the cohort of live births between 2004 and 2013 of children born from mothers residing in the State of São Paulo, having neonatal death (0–27 days) as the main outcome; the cases of congenital anomalies identified and classified in groups; and the causes of death, separated, and without any duplication; and the other variables of interest collected and available in the Live Birth or Death Certificates (Table 3).

## Discussion

The present study described the steps for ensuring the adequacy of secondary data on live births and infant deaths for use in research on neonatal mortality and identified 27 operational challenges of this process. To accomplish this task, 22 procedures and 23 actions were needed to face the challenges encountered, organized in 6 steps. The steps of data preparation and data understanding were identified as the most labor-intensive tasks.

These two steps are those that demand human resources with specific skills and appropriate computational resources to support operations for manipulating databases. In fact, knowledge of the health care system, the background and processes involved in the creation of the data, an understanding of the content, and the ability to think in terms of data structures are essential skills to deal with the data preparation and challenges almost always encountered in the procedures described in the study. And, above all, sufficient human resources must be available for working with these data for the reasons mentioned. Coeli et al. proposed to address the following issues in the training of human resources to work with secondary data: “SQL (Structured Query Language), linking of records, integration of unstructured data, data mining and computational modeling of complex systems” (31).

According to a Brazilian study that proposed to create the National Health Database Centered on the individual, using administrative and epidemiological databases (2000–2015) from four DATASUS Information Systems, cleaning and standardizing data “are relevant and laborious tasks, given the high frequency of inconsistent, incomplete or misspelled data” (3). Our results corroborate the findings of this study, once cleaning and standardizing data are data preparation tasks (step 4), the most labor-intensive step assessed by the present study (3).

No problems were encountered that would result in unsuitable data for the defined use. However, challenges faced at any step may impact the research results. Potential challenges at each step are further outlined and discussed below.

Initially, in step 1 “problem understanding,” the periodic meetings of the working group made it possible to align the technical language between professionals from different areas, minimizing possible communication failures and, consequently, facilitating consensual decision-making. The lack of clarity in defining the needs for using the data can lead to rework, especially in the data preparation step, impacting the time and cost to carry out the research project, in addition to the inadequate sizing up of resources (2). This reinforces the importance of integrating the group to reach the final product, that is, a suitable and simple database file. The success in executing the procedures and actions to face the operational challenges at this step is also due to the availability of data at the beginning of the process, which made it possible to analyze the technical feasibility of the preparation of a database suitable for the intended use and opened a more sustained path for the execution of the next steps. Ethical issues must always permeate the use of secondary health data: every individual has the right to confidentiality, secrecy, and privacy of personal health information, regardless of the medium in which it circulates. Thus, in steps 1 and 3, the recognition of these issues is essential to avoid the misuse of personal information in research (32).

In step 2 “resource planning,” the hardware and software resources existing in the institution proposing the research project were first allocated, avoiding unnecessary expenses. As available human resources, only members of the research group were considered. The choice of software was based on the characteristics of the data sources; the estimates of the volume of input and output data; the data delivery format for the intended use; the functionalities of the tools for data processing and delivery; the budget limits of the research project; and the researchers' experience with the use of resources, aiming to minimize the execution time for data adequacy and avoid errors in the entire process.

Step 3 “data understanding” started with obtaining the secondary data files for the research institution and ended with the data extraction plan. The use of a Relational Database Management Systems (RDBMS) enabled the exploration of around 6 million records distributed in multiple files. Despite a large amount of data, the commands in SQL were executed with good performance in the specified equipment with 16 Gb of RAM, in an interactive way to visualize the data (33). Besides exploring the data content and filling patterns, and consulting the variable dictionaries and operational manuals, the discussions in the multidisciplinary working group facilitated the understanding of the data. Often, the major impediment to the use of secondary data is the unavailability of dictionaries of variables or difficulty in accessing them, which makes the task of understanding these data very arduous. Thus, the researcher's unclarified questions about the data can impair its use. Data cannot be used without an understanding of what it represents and how it was generated, as this can result in errors of interpretation, impacting the results of the research. So, those responsible for the custody of databases must make abundant documentation about their data (31). Moreover, the lack of variability and incomplete filling of variables can make the dataset unfeasible.

RDBMS are suitable for operation with structured data. For steps 3 and 4, an Open Source alternative for MS® SQLServer® is MySQL (33, 34). As an alternative to SQL embedded in any RDBMS, there are also other programming languages, such as Python (35), data extraction, transformation and loading tools, and statistical packages such as R (36) or spreadsheets. The programming language Python (35) or the statistical package R (36) can also be considered as resources for both data preparation and analysis. Spreadsheets are simpler tools and help to manipulate the data but may not support large volumes of them. Whichever tool is chosen for the treatment of the data, it is important to observe its limits regarding the number of records in the rows and variables in the columns; the ease in carrying out the joining, union, and grouping operations; and the performance of the software and the hardware. In short, the most important is that the software should be used according to the intended goal. RDBMS are very useful to prepare data but very inconvenient for statistical analysis. Nevertheless, the more analysis-oriented programs are quite capable of working with relational data structures.

Step 4 was the most critical for the use of the secondary data. Despite this, all the procedures and actions to face the challenges performed in this step depended on the decisions and information obtained in step 1, the resources planned in step 2, and the understanding of the data made in step 3. Prepare the data without prior understanding of them, and the needs of their use can introduce errors that will be propagated to the data analysis phase, generating invalid results. In this step, it is important to check if the transformations were successful, comparing or crossing the original values with the transformed ones, because, when a valid transformation rule to correct a format is identified, there may still be exceptions.

The adequacy of the data was limited to the questions provided in step 1. It is worth mentioning that the planned data transformations for one data set may not be valid for another. New challenges require other coping actions. It is likely that, in the analysis process for which the data was prepared, new unforeseen issues may arise and, therefore, new needs for organization or transformation of an existing database. Although it is not possible to foresee these future needs, the treatment of the data up to this point has allowed them to be understood, cleaned, standardized, consolidated, and reorganized in a structure that facilitates future data transformations, if necessary.

In step 5 “data validation,” the data were subjected to a critical assessment about the content and format of the variables. The data on live births were compared with the annual totals presented on the website of the supplier, the SEADE Foundation, considering the same definition of the study population: residents in the State of São Paulo (26, 30). The similarity between the annual distributions was verified using variables of live births and neonatal deaths. Errors detected in this step may imply the need to return to the steps of understanding and preparing data. In the absence of a reliable source for data validation, it is suggested that the distribution patterns of the variable be observed according to a dimension of time and space and possible outliers and discrepancies are identified.

In step 6 “data distribution,” concerning the output data distribution formats, “*.csv”* (values separated by commas) stands out, due to the possibility of being imported to other platforms and statistical packages, ensuring data portability. In the other formats, “*.sav”* and “*.dat,”* the data and the dictionary of variables are arranged in the same file, preventing the documentation from being lost (37). The other file formats are a proprietary format, developed and maintained as part of each statistical software application, thus there is no guarantee that they can be read by other software. The software manuals must be consulted to import and export files saved in different formats.

The choice of the *OwnCloud* data distribution platform avoided additional costs and ensured data availability for the entire team. After distribution to the group of researchers, the research database was considered ready for use. In this case, the evaluation was made by the same group that participated in the specifications defined in step 1.

Finally, at the end of the data adequacy process, the data were considered understood, integrated, standardized, prepared to meet the requirements, validated, and accessible to researchers on the platform and in the specified formats.

The procedures of this study, described abstractly and chronologically ordered, corresponded to the planned actions and guided the execution of the process. The “operational challenges” and “coping actions,” also described abstractly, corresponded to the unforeseen obstacles and the strategies for their solution, aiming at the fulfillment of the procedures. The result of each step was described in a pragmatic way to reduce the gap between the abstract and theoretical, and the concrete and practical elements. Similar to the checklists or “scientific writing guides,” this work is potentially a proposal for guidance on research work in the health field based on secondary data because it elucidates the challenges in the steps before data analysis and addresses issues that can contribute to the orientation of researchers (38).

As the main limitation of this study, it can be considered that other data sets may present additional challenges that have not been identified. Thus, all results are limited to the data set used as material and the purpose of that research, for which the data were prepared. For example, more complex challenges can be identified when linking data requires more complex procedures than those faced in this study. On the other side, the challenges encountered in the data adequacy process may be overestimated under the following conditions: when researchers are experienced using data from a given source; when the data maintain the same structure over time; or there is no need to prepare or transform data to answer research questions.

Other study limitations are the challenges in classifying the level of difficulty of a data task and similarly, the time required to undertake a task was not measured. Time is related to cost, and both time and cost can be an obstacle to use the secondary data. Generally, the time depends on the data quality, and the complexity of the data preparation tasks, and the experience of the individuals who handle the data. The knowledge of the time to perform the tasks can help in planning the research.

Regarding the generability of the proposed steps for other secondary data structured in Brazil and other countries, we consider that, although the data appear to be suitable for use, there are two essential steps in this process: step 1 (problem understanding), aiming to understand the use of data and survey of its characteristics; and step 3 (data understanding), in order to collect and understand the meaning and how they are organized. In step 1, there are two main items to observe: the first refers to the potential of secondary data to answer the referred research questions; and the second, to ethical restrictions on the intended use. Step 3 will be necessary, except if the researcher is already completely familiar with the data and there has been no change in the structure or domain of the values of the variables. Data understanding avoids the production of information of bad quality. With regard to Brazilian data, we believe that steps 2, 4, and 5 are also applicable unless data pre-processing has already been done, is maintained by a suitable institution, and there is a data administrator available for consult. For example, combining individual health data from more than one source will not be a simple task, as there is no guarantee that individual health data collected from different systems will share unique key identifiers for individuals (39). In addition, many data may have inconsistencies and other quality problems, as previously mentioned (3). Step 6 is necessary to support a group of researchers who share the same data. In this sense, a framework for the evaluation of secondary data for use in epidemiological research can also help to decide on secondary data use. The framework proposed by Sorensen et al. includes the following items: “(1) integrity of the record of individuals, (2) the accuracy and degree of integrity of the recorded data, (3) the size of the data source, (4) the registration period; (5) accessibility, availability, and cost of data; (6) data format; and (7) possibilities of linking with other data sources (linking of records)” (2).

Secondary health data are relevant material to answer public health questions. Initiatives to provide secondary data previously prepared for general purposes, consolidated and clean, minimize the work of researchers in their preparation for specific purposes, and encourage the use of the data. The data from historical series of health events made available on the Data Science Platform applied to Health (PCDaS) of the Oswaldo Cruz Foundation and other epidemiological data warehouse projects are examples of these initiatives (40).

Non-structured health data are outside the scope of this study, however, challenges and tools to manipulate and integrate them with structured data can be addressed in future studies.

## Conclusions

In summary, this study described the steps and identified the challenges in ensuring the adequacy of structured secondary health data for specific use in quantitative scientific research. Significant challenges were encountered in this process, mainly in the data understanding and data preparation steps. Both steps require specific abilities to deal with the dataset. Nonetheless, all the steps were important to get to the final product, that is, a suitable and simple database file. The results obtained suggest that any need for adjustment due to reorganization, cleaning and correction, creation or transformation of variables, change in the original format, integration with other data, or even the understanding of its content can represent an obstacle to the use of secondary data. The use of the described steps to approach structured secondary data and the knowledge of the potential challenges along the process may contribute to planning health research.

## Data Availability Statement

The data analyzed in this study is subject to the following licenses/restrictions: the data are only partially available for public access. The datasets were provided by SEADE (São Paulo State Data Analysis System Foundation). Requests to access these datasets should be directed to http://produtos.seade.gov.br/produtos/mrc/.

## Ethics Statement

The present study is part of a project on neonatal mortality carried out at Federal University of São Paulo and was approved by the Research Ethics Committee of the institution under opinion 2.580.929 of 08/04/2018.

## Author Contributions

KA, TK, MA, and RG designed the study. KA, PB-P, DC-N, RB, AS, and MK contributed for the execution of the research. KA and TK produced the draft text. KA wrote the full version of the text. All authors revised the paper and approved the final version.

## Conflict of Interest

The authors declare that the research was conducted in the absence of any commercial or financial relationships that could be construed as a potential conflict of interest.

## Abbreviations

CDC: Center for Disease Control and Prevention
CRISP-DM: Cross Industry Standard Process for Data Mining
DATASUS: Informatics Department of the Unified Health System
DC: Death Certificates
IBGE: Brazilian Institute of Geography and Statistics
LBC: Live Birth Certificates
PCDaS: Data Science Platform applied to Health
RDBMS: Relational Database Management Systems
RIPSA: Interagency Health Information Network
SEADE: São Paulo State Data Analysis System Foundation
SIM: Mortality Information System
SINASC: Live Birth Information System
SQL: Structured Query Language.
