# Supplementary material for: Operational Challenges in the Use of Structured Secondary Data for Health Research
Source: Front Public Health. 2021 Jun 15;9:642163. doi: 10.3389/fpubh.2021.642163 (PMC8239175; doi:10.3389/fpubh.2021.642163)
Supplement: Supplementary file 1 [file Table_1.DOCX]

**Supplementary Table 1**. Characteristic of the files of live births and infant deaths and the file of neonatal deaths integrated into the cohort of *live births of mothers living in the State of São Paulo, 2004-2013, made available by the SEADE Foundation*

|  | **Live Births*** | | **Infant Deaths*** | | **Neonatal Deaths^#^** | |
| --- | --- | --- | --- | --- | --- | --- |
| Year of birth | File size in bytes | Nº of records | File size in bytes | Nº of records | Nº of records | Nº . of records integrated into the cohort of live births |
| 2004 | 51,976,894 | 599,652 | 6,536,192 | 8,037 | 5,611 | 5,598 |
| 2005 | 51,468,789 | 619,107 | 6,172,672 | 7,706 | 5,357 | 5,354 |
| 2006 | 49,603,409 | 604,028 | 7,177,728 | 7,845 | 5,415 | 5,389 |
| 2007 | 59,627,263 | 595,449 | 6,075,392 | 7,622 | 5,228 | 4,828 |
| 2008 | 59,332,969 | 601,872 | 5,897,216 | 7,402 | 5,132 | 5,115 |
| 2009 | 58,687,420 | 598,909 | 2,025,804 | 7,522 | 5,192 | 5,184 |
| 2010 | 57,860,731 | 601,561 | 2,516,605 | 6,959 | 4,834 | 4,820 |
| 2011 | 58,461,738 | 610,492 | 5,459,968 | 6,633 | 4,444 | 4,371 |
| 2012 | 55,686,974 | 617,084 | 5,137,408 | 6,962 | 4,846 | 4,808 |
| 2013 | 54,143,572 | 611,300 | 5,556,736 | 7,047 | 4,783 | 4,780 |
| **Total** | **556,849,759** | **6,059,454** | **52,555,721** | **73,735** | **50,842** | **50,247** |

* Input files; #File output in three formats and their sizes: csv (2.3 GB), sav (2.5 GB) e dta (3.3 GB)
