# Supplementary material for: Operational Challenges in the Use of Structured Secondary Data for Health Research
Source: Front Public Health. 2021 Jun 15;9:642163. doi: 10.3389/fpubh.2021.642163 (PMC8239175; doi:10.3389/fpubh.2021.642163)
Supplement: Supplementary file 2 [file Table_2.DOCX]

**Supplementary Table 2**. Dictionary of variables from the final database, containing the data prepared for use.

| Variável | Tipo* | Tamanho | Descrição da variável | Descrição dos valores |
| --- | --- | --- | --- | --- |
| ANO_COORTE | N | 4 | Ano da coorte, de acordo com o informado no banco de dados |  |
| ID | N | 6 | Identificação (sequencial) do registro no momento da importação para este estudo- usar combinado com o ANO_COORTE |  |
| UN1_DTNASC | D | 10 | Data de nascimento |  |
| UN1_HORANASC | T | 4 | Hora de Nascimento da Criança no formato HHMM |  |
| UN1_CODMUNRES_6 | T | 6 | Código do Município de Residência da Criança – 6 dígitos |  |
| UN1_CODMUNRES_7 | T | 7 | Código do Município de Residência da Criança – 7 dígitos |  |
| UN1_UFRES | T | 2 | UF de Residência da Criança |  |
| UN1_CODMUNNASC_6 | T | 6 | Código do Município de Nascimento da Criança – 6 dígitos |  |
| UN1_CODMUNNASC_7 | T | 7 | Código do Município de Nascimento da Criança – 7 dígitos |  |
| UN1_UFNASC | T | 2 | UF de Nascimento da Criança |  |
| UN1_LOCNASC | N | 1 | Local de Nascimento da Criança | 1-Hospital  2-Outro Estab Saúde  3-Domicílio  4-Outros |
| UN1_SEXO | N | 1 | Sexo da Criança | 1-Masculino  2-Feminino  3-Indeterminado |
| UN1_RACACOR | N | 1 | Raça/Cor da Criança (a mesma raça/cor da mãe) | 1-Branca  2-Preta  3-Amarela  4-Parda  5-Indígena |
| UN1_APGAR1 | N | 1 | Índice de Apgar 1º Minuto |  |
| UN1_APGAR5 | N | 1 | Índice de Apgar 5º Minuto |  |
| UN1_PESO | N | 4 | Peso ao Nascer (em gramas) |  |
| UN1_IDADEMAE | N | 2 | Idade da Mãe (em anos) |  |
| UN1_ESCOL_MAE | N | 1 | Escolaridade da Mãe (UNIFICADA) | 1-Nenhum  2-de 1 a 3 anos/fundamental ( 1 a 4 série)  3-de 4 a 7 anos/fundamental ( 5 a 8 série)  4-de 8 a 11 anos/médio (antigo 2 grau)  5-12 anos e mais/superior completo ou incompleto |
| UN1_ESTCIVMAE | N | 1 | Estado Civil da Mãe | 1-Solteira  2-Casada  3-Viúva  4-Separado judicialmente/Divorciado  5-União consensual |
| UN2_IDADEPAI | N | 1 | Idade do pai- informada na DNV |  |
| UN1_GRAVIDEZ | N | 1 | Tipo de Gravidez | 1-Única  2-Dupla  3-Tripla e mais |
| UN1_PARTO | N | 1 | Tipo de Parto | 1-Vaginal  2-Cesáreo |
| UN1_QTDFILMORT | N | 1 | Número de Filhos Nascidos Mortos em Gestações Anteriores |  |
| UN1_QTDFILVIVO | N | 1 | Número de Filhos Nascidos Vivos em Gestações Anteriores |  |
| UN1_CONSULTAS | N | 1 | Consultas de Pré-natal | 1-nenhuma  2-de 01 a 03  3-de 04 a 06  4-de 07 e mais |
| UN1_GESTACAO | N | 1 | Duração da Gestação | 1-Menos de 22 semanas  2-22 a 27 semanas  3-28 a 31 semanas  4-32 a 36 semanas  5-37 a 41 semanas  6-42 semanas e mais |
| UN1_IDANOMAL | N | 1 | Presença de Malformação Congênita e/ou Anomalia Cromossômica | 1-Sim  2-Não |
| UN1_CAN1 | T | 4 | Código da anomalia -1 (CID-10) |  |
| UN1_CAN1_DESCR | T | 255 | Descrição da anomalia -1 (CID-10) |  |
| UN1_CAN2 | T | 4 | Código da anomalia -2 (CID-10) |  |
| UN1_CAN2_DESCR | T | 255 | Descrição da anomalia -2 (CID-10) |  |
| UN1_CAN3 | T | 4 | Código da anomalia -3 (CID-10) |  |
| UN1_CAN3_DESCR | T | 255 | Descrição da anomalia -3 (CID-10) |  |
| UN1_CAN4 | T | 4 | Código da anomalia -4 (CID-10) |  |
| UN1_CAN4_DESCR | T | 255 | Descrição da anomalia -4 (CID-10) |  |
| UN1_CAN5 | T | 4 | Código da anomalia -5 (CID-10) |  |
| UN1_CAN5_DESCR | T | 255 | Descrição da anomalia -5 (CID-10) |  |
| UN1_CAN6 | T | 4 | Código da anomalia -6 (CID-10) |  |
| UN1_CAN6_DESCR | T | 255 | Descrição da anomalia -6 (CID-10) |  |
| UN1_CAN7 | T | 4 | Código da anomalia -7 (CID-10) |  |
| UN1_CAN7_DESCR | T | 255 | Descrição da anomalia -7 (CID-10) |  |
| UN1_CAN8 | T | 4 | Código da anomalia -8 (CID-10) |  |
| UN1_CAN8_DESCR | T | 255 | Descrição da anomalia -8 (CID-10) |  |
| UN1_CAN9 | T | 4 | Código da anomalia -9 (CID-10) |  |
| UN1_CAN9_DESCR | T | 255 | Descrição da anomalia -9 (CID-10) |  |
| UN1_CAN10 | T | 4 | Código da anomalia -10 (CID-10) |  |
| UN1_CAN10_DESCR | T | 255 | Descrição da anomalia -10 (CID-10) |  |
| UO_DTOBITO | D | 10 | Data de óbito |  |
| UO_HORAOBITO | T | 4 | Hora do óbito da Criança no formato HHMM |  |
| X_UO_IDADE | N | 1 | Idade (original), no formato Código da Idade + Valor da Idade (0:Minutos,1:Horas,2:Dias,3:Meses,4:Anos,5:100 Anos ou mais,9:Ignorado) |  |
| UO_IDADE_EM_HORAS | F | 8.3 | Idade do falecido em horas ** |  |
| UO_UFRES | T | 6 | UF de Residência da Criança - informado na DO |  |
| UO_LOCOCOR | N | 1 | Local de Ocorrência do óbito | 1-Hospital  2-Outro Estab Saúde  3-Domicílio  4-Outros |
| UO_CODMUNOCOR_7 | T | 7 | Código do Município de Ocorrência do óbito da Criança – 7 dígitos - informado na DO |  |
| UO_CODMUNOCOR_6 | T | 6 | Código do Município de Ocorrência do óbito da Criança – 6 dígitos - informado na DO |  |
| UO_UFOCOR | T | 2 | UF de Ocorrência do óbito - informado na DO |  |
| UO_ASSISTMED | N | 1 | Recebeu Assistência Médica | 1-sim  2-não |
| UO_EXAME | N | 1 | Indica se houve exame complementar | 1-sim  2-não |
| UO_CIRURGIA | N | 1 | Indica se houve cirurgia | 1-sim  2-não |
| UO_NECROPSIA | N | 1 | Indica se houve necropsia | 1-sim  2-não |
| UO_ATESTANTE | N | 1 | Médico que Assina o Atestado de Óbito | 1-Médico que atendeu o paciente durante a doença que ocasionou o óbito  2-Médico Substituto  3-IML  4-SVO  5-Outros |
| UO_CIRCOBITO | N | 1 | Circunstâncias do Óbito (morte não natural) | 1-Acidente  2-Suicídio  3-Homicídio  4-Outros |
| UO_FONTE | N | 1 | Fonte da informação | 1-Boletim de Ocorrência  2-Hospital  3-Família  4-Outra |
| UO_NEONATAL | T | 1 | Óbito neonatal | 0-não  1-sim |
| UO_OBITO_ANOMALIA | N | 1 | Óbito por anomalia em qualquer posição |  |
| UN1_UO_ANOMALIA | N | 1 | Anomalia no nascimento ou no óbito | 0-não é caso de anomalia no nascimento nem no óbito  1-é caso de anomalia - morreu por anomalia ou nasceu com anomalia |
| UO_CAUSABAS | T | 4 | Causa básica do óbito (CID-10) |  |
| UO_CAUSABAS_DESCR | T | 255 | Descrição da causa básica de óbito ( CID-10). |  |
| A1 | T | 4 | Causa Mencionada na Linha A – 1ª. Posição – CID10 |  |
| A1_DESCR | T | 255 | Descrição da Causa Mencionada na Linha A – 1ª. Posição – CID10 |  |
| A2 | T | 4 | Causa Mencionada na Linha A – 2ª. Posição – CID10 |  |
| A2_DESCR | T | 255 | Descrição da Causa Mencionada na Linha A – 2ª. Posição – CID10 |  |
| A3 | T | 4 | Causa Mencionada na Linha A – 3ª. Posição – CID10 |  |
| A3_DESCR | T | 255 | Descrição da Causa Mencionada na Linha A – 3ª. Posição – CID10 |  |
| A4 | T | 4 | Causa Mencionada na Linha A – 4ª. Posição – CID10 |  |
| A4_DESCR | T | 255 | Descrição da Causa Mencionada na Linha A – 4ª. Posição – CID10 |  |
| A5 | T | 4 | Causa Mencionada na Linha A – 5ª. Posição – CID10 |  |
| A5_DESCR | T | 255 | Descrição da Causa Mencionada na Linha A – 5ª. Posição – CID10 |  |
| B1 | T | 4 | Causa Mencionada na Linha B – 1ª. Posição – CID10 |  |
| B1_DESCR | T | 255 | Descrição da Causa Mencionada na Linha B – 1ª. Posição – CID10 |  |
| B2 | T | 4 | Causa Mencionada na Linha B – 2ª. Posição – CID10 |  |
| B2_DESCR | T | 255 | Descrição da Causa Mencionada na Linha B – 2ª. Posição – CID10 |  |
| B3 | T | 4 | Causa Mencionada na Linha B – 3ª. Posição – CID10 |  |
| B3_DESCR | T | 255 | Descrição da Causa Mencionada na Linha B – 3ª. Posição – CID10 |  |
| B4 | T | 4 | Causa Mencionada na Linha B – 4ª. Posição – CID10 |  |
| B4_DESCR | T | 255 | Descrição da Causa Mencionada na Linha B – 4ª. Posição – CID10 |  |
| B5 | T | 4 | Causa Mencionada na Linha B – 5ª. Posição – CID10 |  |
| B5_DESCR | T | 255 | Descrição da Causa Mencionada na Linha B – 5ª. Posição – CID10 |  |
| C1 | T | 4 | Causa Mencionada na Linha C – 1ª. Posição – CID10 |  |
| C1_DESCR | T | 255 | Descrição da Causa Mencionada na Linha C – 1ª. Posição – CID10 |  |
| C2 | T | 4 | Causa Mencionada na Linha C – 2ª. Posição – CID10 |  |
| C2_DESCR | T | 255 | Descrição da Causa Mencionada na Linha C – 2ª. Posição – CID10 |  |
| C3 | T | 4 | Causa Mencionada na Linha C – 3ª. Posição – CID10 |  |
| C3_DESCR | T | 255 | Descrição da Causa Mencionada na Linha C – 3ª. Posição – CID10 |  |
| C4 | T | 4 | Causa Mencionada na Linha C – 4ª. Posição – CID10 |  |
| C4_DESCR | T | 255 | Descrição da Causa Mencionada na Linha C – 4ª. Posição – CID10 |  |
| C5 | T | 4 | Causa Mencionada na Linha C – 5ª. Posição – CID10 |  |
| C5_DESCR | T | 255 | Descrição da Causa Mencionada na Linha C – 5ª. Posição – CID10 |  |
| D1 | T | 4 | Causa Mencionada na Linha D – 1ª. Posição – CID10 |  |
| D1_DESCR | T | 255 | Descrição da Causa Mencionada na Linha D – 1ª. Posição – CID10 |  |
| D2 | T | 4 | Causa Mencionada na Linha D – 2ª. Posição – CID10 |  |
| D2_DESCR | T | 255 | Descrição da Causa Mencionada na Linha D – 2ª. Posição – CID10 |  |
| D3 | T | 4 | Causa Mencionada na Linha D – 3ª. Posição – CID10 |  |
| D3_DESCR | T | 255 | Descrição da Causa Mencionada na Linha D – 3ª. Posição – CID10 |  |
| D4 | T | 4 | Causa Mencionada na Linha D – 4ª. Posição – CID10 |  |
| D4_DESCR | T | 255 | Descrição da Causa Mencionada na Linha D – 4ª. Posição – CID10 |  |
| D5 | T | 4 | Causa Mencionada na Linha D – 5ª. Posição – CID10 |  |
| D5_DESCR | T | 255 | Descrição da Causa Mencionada na Linha D – 5ª. Posição – CID10 |  |
| D6 | T | 4 | Causa Mencionada na Linha D – 6ª. Posição – CID10 |  |
| D6_DESCR | T | 255 | Descrição da Causa Mencionada na Linha D – 6ª. Posição – CID10 |  |
| II_1 | T | 4 | Causa Mencionada na Linha II – 1ª. Posição – CID10 |  |
| II_1_DESCR | T | 255 | Descrição da Causa Mencionada na Linha II – 1ª. Posição – CID10 |  |
| II_2 | T | 4 | Causa Mencionada na Linha II – 2ª. Posição – CID10 |  |
| II_2_DESCR | T | 255 | Descrição da Causa Mencionada na Linha II – 2ª. Posição – CID10 |  |
| II_3 | T | 4 | Causa Mencionada na Linha II – 3ª. Posição – CID10 |  |
| II_3_DESCR | T | 255 | Descrição da Causa Mencionada na Linha II – 3ª. Posição – CID10 |  |
| II_4 | T | 4 | Causa Mencionada na Linha II– 4ª. Posição – CID10 |  |
| II_4_DESCR | T | 255 | Descrição da Causa Mencionada na Linha II – 4ª. Posição – CID10 |  |
| II_5 | T | 4 | Causa Mencionada na Linha II – 5ª. Posição – CID10 |  |
| II_5_DESCR | T | 255 | Descrição da Causa Mencionada na Linha II – 5ª. Posição – CID10 |  |
| II_6 | T | 4 | Causa Mencionada na Linha II – 6ª. Posição – CID10 |  |
| II_6_DESCR | T | 255 | Descrição da Causa Mencionada na Linha II – 6ª. Posição – CID10 |  |
| G1_OBITO_ANOMALIA | N | 1 | Morte por anomalia Grupo 1- Q00 a Q07 | 0 “não”  1 “sim” |
| G2_OBITO_ANOMALIA | N | 1 | Morte por anomalia Grupo 2- Q10 a Q18 | 0 “não”  1 “sim” |
| G3_OBITO_ANOMALIA | N | 1 | Morte por anomalia Grupo 3- Q20 a Q28 | 0 “não”  1 “sim” |
| G4_OBITO_ANOMALIA | N | 1 | Morte por anomalia Grupo 4- Q30 a Q34 | 0 “não”  1 “sim” |
| G5_OBITO_ANOMALIA | N | 1 | Morte por anomalia Grupo 5- Q35 a Q37 | 0 “não”  1 “sim” |
| G6_OBITO_ANOMALIA | N | 1 | Morte por anomalia Grupo 6- Q38 a Q45 | 0 “não”  1 “sim” |
| G7_OBITO_ANOMALIA | N | 1 | Morte por anomalia Grupo 7- Q50 a Q56 | 0 “não”  1 “sim” |
| G8_OBITO_ANOMALIA | N | 1 | Morte por anomalia Grupo 8- Q60 a Q64 | 0 “não”  1 “sim” |
| G9_OBITO_ANOMALIA | N | 1 | Morte por anomalia Grupo 9- Q65 a Q79 | 0 “não”  1 “sim” |
| G10_OBITO_ANOMALIA | N | 1 | Morte por anomalia Grupo 10- Q80 a Q89 | 0 “não”  1 “sim” |
| G11_OBITO_ANOMALIA | N | 1 | Morte por anomalia Grupo 11- Q90 a Q99 | 0 “não”  1 “sim” |

| Note: all variables went through the cleaning / consistency process and had their invalid values eliminated (converted to “missing value”). |
| --- |
| The variables with suffix _DESCR were added to facilitate the interpretation of the code |
| * Type: N = numeric; T = text; D = date; F = numeric with decimal places (“float”). |

**CONGENITAL ANOMALY**

Neonatal deaths with some congenital anomaly were classified according to the table below. Eleven dichotomous variables were created, 1 for each of the groups, since the same individual may present anomalies that are classified in more than one group.

<https://www.prefeitura.sp.gov.br/cidade/secretarias/upload/saude/arquivos/publicacoes/Manual_DN_02fev2011.pdf>
